# Obesity and Multiple Sclerosis—A Multifaceted Association

**DOI:** 10.3390/jcm10122689

**Published:** 2021-06-18

**Authors:** Thomas-Gabriel Schreiner, Tudor-Marcel Genes

**Affiliations:** 1 Neurology Department, Clinical Rehabilitation Hospital, 700661 Iași, Romania; genestudor@yahoo.com; 2 Faculty of Medicine, University of Medicine and Pharmacy “Gr. T. Popa”, 700115 Iași, Romania; 3 Faculty of Medicine, University of Medicine and Pharmacy “Carol Davila”, 050474 Bucharest, Romania

**Keywords:** multiple sclerosis, obesity, pathophysiology, chronic inflammation, adipokines

## Abstract

Background: Given the common elements in the pathophysiological theories that try to explain the appearance and evolution of obesity and multiple sclerosis, the association between the two pathologies has become an increasingly researched topic in recent years. On the one hand, there is the chronic demyelinating inflammation caused by the autoimmune cascade of multiple sclerosis, while on the other hand, according to the latest research, it has been shown that obesity shares an inflammatory component with most chronic diseases. Methods: The authors performed independent research of the available literature in the most important electronic databases (PubMed, Google Scholar, Embase, and Science Direct) in February 2021. After applying the exclusion criteria, the reviewers focused on the most relevant articles published during the last 10 years with respect to epidemiology and pathophysiology. Results: The data presented are a step forward in trying to elucidate the intricate relationship between obesity and MS, especially the causal relationship between childhood and adolescent obesity and MS, focusing on the epidemiological associations observed in the most relevant observational studies conducted in recent years. In the second part, the authors comment on the latest findings related to the pathophysiological mechanisms that may explain the correlations between obesity and multiple sclerosis, focusing also on the role of adipokines. Conclusions: Based on available epidemiological data, obesity in early life appears to be strongly associated with a higher risk of MS development, independent of other risk factors. Although much research has been done on the pathophysiology of obesity, MS, their possible common mechanism, and the role of adipokines, further studies are needed in order to explain what remains unknown. No relevant data were found regarding the association between obesity, disability (high EDSS score), and mortality risk in MS patients. Thus, we consider that this topic should be elucidated in future research.

## 1. Introduction

### 1.1. Multiple Sclerosis—A Global Burden

Multiple sclerosis (MS) is the most common chronic inflammatory disease of the central nervous system (CNS), with an increasing incidence, currently affecting over 2 million people worldwide (Bezzini and Battaglia, 2017) [1]. MS is characterized by demyelinating lesions that produce typical but also nonspecific neurological symptoms: monocular visual loss due to optic neuritis, sensory loss and muscle weakness due to transverse myelitis, cranial nerves pathology in brainstem dysfunction, or ataxia due to important cerebellar lesions, which cause disability and declining quality of life, especially in professionally active young people (Oh et al., 2018) [2]. Given the important socio-economic impact, MS is still extensively studied, and although there are many therapeutic options that can slow the progression of the disease and reduce the number of relapses, there is currently no curative treatment. Therefore, it is of utmost importance to fully understand the pathophysiology (autoimmune cascade) and detect risk factors, with increasing emphasis on environmental factors such as climate, stress, viral infections (i.e., EBV), smoking, low concentrations of vitamin D, eating habits (Western lifestyle), and obesity (Olsson et al., 2017) [3].

### 1.2. Obesity as a Public Health Problem

Obesity and obesity-related pathologies have significantly increased in prevalence, for both sexes and for all age groups, including children and young adults, in both well-developed and many developing countries around the world (Jaacks et al., 2019) [4]. In Romania, for example, 19.1% of the adult population was obese in 2010. The prevalence of obesity was higher among women: the proportion of men who were obese was 16.9% compared to 21.2% in women. In the pediatric population, the impact of obesity is also significant: up to 33% of boys and 19% of girls among 11-year-olds were overweight, according to data from the School Behavior in Health (HBSC) survey (2009/2010). For other age groups, the corresponding figures were 25% for 13-year-olds boys and 15% for girls, and among 15-year-olds, 27% and 10%, respectively (WHO Europe, 2013) [5].

Two measurement methods objectify the obese status of an adult. The first is BMI, a somatometric variable that defines overweight at values between 25.0 and 29.9 kg/m^2^ and obesity at values of 30 kg/m^2^ or more. A complementary diagnostic criterion for the definition of obesity in adults is the measurement of abdominal (waist) circumference, which is performed by placing the tailoring meter in the middle of the distance between the lower edge of the last rib and the upper edge of the iliac crest, with the patient in standing position and complete expiration. Abdominal adiposity is defined by a value of more than 88 cm (35 inches) in women and 102 cm (40 inches) in men (Ross et al., 2020) [6]. This method is mainly used in the diagnosis of metabolic syndrome (MetS), which is associated with cardiovascular risk factors and predisposes one to cardio- and neurovascular diseases (Swarup et al., 2020) [7]. Multiple studies have shown the correlation between these two measurement methods, and current guidelines recommend performing both measurements pertaining to the type of obesity (abdominal). In the case of children, it is recommended to use a scale of percentiles based on the sex and age of the child. In this population, overweight is defined as a BMI in the 85th to 94th percentile, and obesity as a BMI at or above the 95th percentile (Weir and Jan, 2020) [8]. From a pathophysiological point of view, recent studies suggest the character of chronic inflammation in adipose tissue, with an accentuated inflammatory condition in the case of obesity. In addition, epidemiological data show the association between obesity and other chronic diseases with an important inflammatory component such as chronic kidney disease, inflammatory bowel disease, psoriasis (Kumthekar and Ogdie, 2020) [9], and neurological pathology such as multiple sclerosis.

### 1.3. Aim of the Review

The main purpose of this review is to detail the intricate association between obesity and MS. Because it was initially considered only as an epidemiological correlation, the first part of the paper presents the most relevant and recent data on this topic. Discussing the case of both adult and pediatric MS, obesity seems to be not only an environmental risk factor, as it is also thought to play a significant role in the evolution of the disease. Thus, in the second part, the emphasis is on the elements of pathophysiology, with a focus on the currently accepted theories, including the role of adipokines.

## 2. Material and Methods

**Figure 1:**
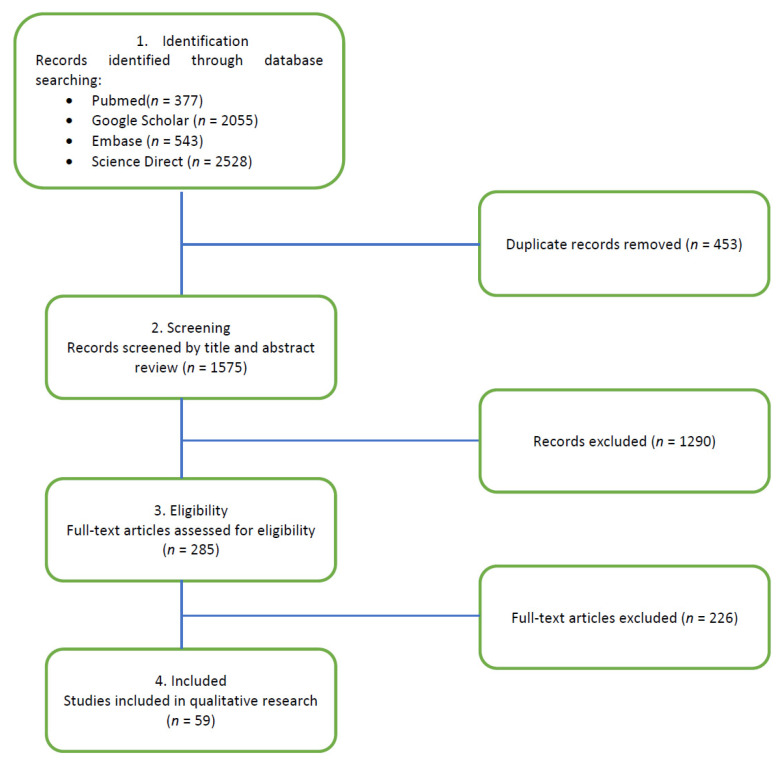
PRISMA flow diagram.

In order to achieve the aim of this article, the most important electronic databases (PubMed, Google Scholar, Embase, and Science Direct) were searched for articles published in the English language mostly from 1 January 2010 to the present involving human subjects, but older articles were also included in this review if relevant. The two authors conducted independent searches in February 2021 using the following combinations of terms:

- for the epidemiology part of the review: “multiple sclerosis” AND (“obesity” OR “overweight” OR “overnutrition”) AND/OR “epidemiology”;
- for the second part regarding the pathophysiology: “multiple sclerosis” AND (“obesity” OR “overweight” OR “overnutrition”) AND/OR “inflammation” AND/OR (“physiology” OR “pathophysiology”);

After applying the exclusion criteria (non-English language, abstract-only articles, animal research studies, opinion-based letters-to-the-editors, and non-MS trials), the reviewers focused on the most relevant articles published during the last 10 years with respect to epidemiology and pathophysiology (see Figure 1). 

## 3. Obesity as an Environmental Risk Factor in Multiple Sclerosis

### 3.1. Obesity and Multiple Sclerosis in Adults

Studies on large cohorts of patients have been conducted over the last decade to detect and demonstrate the involvement of environmental risk factors in MS. Two relevant studies that have paved the way for further research on this topic are worth mentioning. Munger et al., 2013, showed an epidemiological correlation between adolescent obesity and the risk of developing MS in women [10]. The second study, the one conducted by Alfredsson and Olsson, 2019, expanded the research and demonstrated that regardless of gender, childhood and adolescence obesity doubles the risk of incidence for MS, with BMI > 27 kg/m^2^ more strongly correlated with the disease [11]. In addition, other studies have estimated with remarkable accuracy the age at which obesity is most likely to be a risk factor for MS. Specifically, according to Hedstrom et al., 2016, the critical period seems to be during adolescence and not during childhood (the first years of life) [12]. 

In order to have the best possible accuracy of the epidemiological findings and to avoid bias, Mendelian randomization studies followed. The aim was to show that genetic determinants for high BMI are associated with an increased risk of MS. The study led by Mokry et al., 2016, revealed the direct association between an increased genetic BMI and the risk of MS, with obesity playing a causal role in the etiology of MS. While it was already known that obesity is a risk factor for many end-of-life pathologies (especially cardiovascular pathology), the study also suggests an important consequence of obesity in childhood and/or early adulthood [13].

One of the most recent research studies on the topic is the Mendelian randomization study conducted by (Harroud et al., 2021). Including large cohorts of MS and control cases, authors showed that higher childhood BMI is associated with agreater risk of MS, independent of age at puberty [14]. Thus, besides environmental factors, genetic evidence also confirms the influence of childhood obesity on MS susceptibility.

### 3.2. Obesity and Pediatric MS

In recent years, similar to research on environmental risk factors in adult MS, research on MS with pediatric onset has multiplied.

A study worth mentioning that generated a lot of discussion at the time of its emergence is the research of Langer-Gould et al., 2013. According to the results, obesity was associated with a significantly increased risk of MS/CIS (clinically isolated syndrome) in girls but not in boys. The adjusted probability ratio and 95% confidence intervals for CIS/MS among girls were 1.58 (0.71–3.50) for overweight compared to normal weight (reference category), 1.78 (0.70–4.49) for moderate obesity, and 3.76 (1.54–9.16) for extremely obese. A direct proportionality is observed between the severity of obesity and the risk of disease. Moreover, patients with moderate and severe obesity were more likely to have transverse myelitis compared to normal/overweight children. It is suggested that the childhood obesity epidemic is likely to increase morbidity in MS/CIS, especially in adolescents [15].

In addition to the previous observations, the work of Chitnis et al., 2016, illustrates the relative contributions of body mass index (BMI) and pubertal measures (Tanner stages) for the risk and age of onset of pediatric MS. According to the study, the onset of prepubertal MS (Tanner stage I) is rare, found in only 11% of girls and 15% of boys included in the trial, while 80% of girls presented MS after menarche. In terms of obesity, high BMI in early adolescence is a risk factor for MS in girls and boys. Earlier age at sexual maturity contributes to an earlier age at the onset of MS, especially in association with obesity [16]. A year later, Cappa et al., 2017, identify through their results that many of the risk factors for MS in children are the same as in adult MS. It has been shown that childhood obesity is independently associated with an increased risk of pediatric MS and CIS in girls (but not in boys), and this risk is dose-dependent. In addition, obesity before the age of 20 is an independent risk factor for MS in adults [17].

Huppke et al., 2019, went a step further by studying the effect of obesity on the evolution of the disease in children. A total of 27.8% of the children included in the study were overweight or obese, and obesity was associated with statistically significant double chances of MS in both sexes. Obese patients, compared to normal-weight patients, had statistically significantly more recurrences in first-line treatment with interferon beta and glatiramer acetate. However, the initial neuroimaging, the interval between the first and second MS attack, pretreatment relapses, and EDSS (Expanded Disability Status Scale) progression scores were not correlated with BMI [18].

This study is in line with an older study on adult MS conducted by Kvistad et al., 2015, on a small group (86 patients). The authors observed a correlation between obesity and increased disease activity during interferon beta therapy, according to the NEDA (no evidence of disease activity) evaluation. This may indicate that BMI affects the response to interferon-beta treatment. No difference in disability was observed as measured by the EDSS scale in the obese group compared to the normal weight [19]. 

The Mendelian randomization study led by Gianfrancesco et al., 2017, reports strong evidence for a causal and independent association between low serum vitamin D levels, increased BMI, and the risk of MS in children and adolescents; their results were obtained after adjusting for factors that could cause bias, such as sex, ancestors, and over 100 non-human leukocyte antigens (HLA) risk variants for MS. Moreover, the unclear association from previous studies that attributed to an increased risk of MS associated with BMI to the lower levels of vitamin D observed in obese individuals is not demonstrated. The findings of the work of Gianfrancesco et al., 2017 support an independent contribution of low vitamin D level to disease susceptibility [20].

The data discussed above are summarized in Table 1.

However, not all studies show a causal relationship between obesity and MS. For example, in the study conducted by Pinhas-Hamiel et al., 2015, on a cohort of patients aged 40–65 years, disease duration > 10 years and neurological disability according to the EDSS score > 3, adult patients with MS with disabilities had lower rates of obesity and overweight, according to the BMI assessment, compared to the general population. At the same time, the prevalence of MetS was similar to that of the general population. Higher rates of increased waist circumference were found, suggesting that lower BMI may be misleading in terms of health risk [21].

### 3.3. Geographical Area as Influence for the Obesity–MS Correlation

The environmental risk factors for MS are diverse, and their action can be intricate. The influence of culture/geographical area in the epidemiological equation must not be forgotten, and this aspect playings a role in both obesity and in the epidemiology of MS. Four cohort studies from different geographic areas are relevant to this topic, and the main conclusions and statistical parameters are presented in Table 2.

Although there are small variations in incidence and prevalence among the mentioned studies, most of them showed a significant positive association between obesity and MS, regardless of the presence or absence of other environmental factors. Furthermore, data from preclinical trials support the epidemiological findings: recent work conducted on the experimental autoimmune encephalomyelitis (EAE) mouse model by Ji et al., 2019, suggests that obesity induces EAE through IL-6 upregulation that mediates T cell activation and CNS infiltration [26]. Similarly, diet-induced obesity in mice upregulates specific genes that play a significant role in inducing and sustaining EAE, explaining the genetic component of obesity in MS (Hasan et al., 2016) [27].

## 4. Common Pathophysiological Elements for Obesity and Multiple Sclerosis

Research in recent years has attempted to shed light on the pathophysiology of the aforementioned epidemiological associations between obesity and MS. From a pathophysiological and immunological point of view, MS and obesity have in common at least three distinctive pathways that are not mutually exclusive but may overlap (Alfredsson and Olsson, 2019) [11].

### 4.1. The Inflammatory Theory

Inflammatory theory in MS is well known and demonstrated, with this neurological disease being initially characterized by a chronic inflammation that in the late stages is replaced by neurodegeneration (Giovannoni, 2017) [28]. At the same time, the latest studies show that obesity, which involves hypertrophy and subsequent adipocyte hyperplasia, is characterized by a “low-grade” inflammation in which high levels of proinflammatory mediators occur in the adipose tissue. Physiologically, in addition to the storage function, fat cells secrete many endogenous substances, either hormones, such as leptin, adiponectin, and resistin, or cytokines, such as IL-6 or TNFalpha. In the case of obesity, there is a hypersecretion of cytokines that will excessively stimulate the recruitment of immune cells (Asghar and Sheikh, 2017) [29].

The trigger for this inflammation is not fully known, but it is suspected to be associated with stress due to excess energy that destabilizes the homeostatic balance. Although the initial inflammatory responses appear to be adaptive by reducing anabolic pressure, they still unbalance homeostasis. As it progresses, chronic low-grade inflammation sets new benchmarks for blood sugar, lipidemia, hormones, or vascular tone, causing, for example, resistance to insulin and explaining the occurrence of other components of MetS (Karczewski et al., 2018) [30].

In addition, in adipose tissue other than mature adipocytes, there are pre-adipocytes and immune cells whose functions are altered in obese people. Thus, the increased number of pre-adipocytes determines insulin resistance, while hypersecretion of IL-6 reduces at the pre-adipocyte level the secretion of resistine, adiponectin, glucose-4 transporter (GLUT-4) and the expression of the insulin-1 (IRS-1) receptor substrate. Moreover, TNF-alpha stimulates the secretion of MCP-1 and IL-6 from pre-adipocytes, supporting pro-inflammatory status (Sarantopoulos et al., 2018) [31].

Regarding immune cells, the abnormal behavior of lymphocytes is of interest for the association of obesity–MS-altered immune response. In the case of obesity, there is a significant change in the number of Th1 cells and Th2 cells, in the sense of a decrease in Th2 and an increase in Th1, which is similar to the situation encountered in MS. Th17, another immunological marker studied in the context of MS (Balasa et al., 2020) [32], is increased in obesity and is thought to be responsible for inducing insulin resistance by influencing JNK (Jun-NH2 terminal kinases). Moreover, it is accompanied by a decrease in the number of T reg cells with an anti-inflammatory effect similar to MS. Upregulation of CD8 T cells is another important change in obesity that explains insulin resistance, more recently proving to play an important role in MS (Raud et al., 2018) [33]. 

It has also been observed in relation to lymphocytes that the number of B cells increases with obesity, while the decrease in their number is associated with a decrease in insulin resistance. Moreover, it has been shown that B cell-derived IgG2c immunoglobulin levels are elevated in obesity and experimentally introduced immunoglobulin in normal-weight subjects causes inflammation of adipose tissue and insulin resistance with stimulated T cells (McLaughlin et al., 2017) [34]. In the pathogenesis of MS, the role of B cells has recently become a central subject of study, with different theories suggesting that antibody-dependent and antibody-independent factors initiate inflammatory demyelination, either by forming specific autoantibodies for CNS structures that will activate T cells, or by the formation of ectopic lymphoid structures producing cytokines and neurotoxic factors (Wanleenuwat and Iwanowski, 2019) [35].

### 4.2. The Hormonal Theory—Focus on the Role of Adipokines as Biomarkers in Obese Patients with MS 

The second theory is the hormonal theory in obesity, in which excess adipose tissue is accompanied by a disturbed secretion of adipokines. 

Adipose tissue is not just an energy storage tissue but a true active endocrine organ that secretes cytokines. Called adipokines, these are both pro- and anti-inflammatory mediators, with leptin, adiponectin, and resistin among the most studied. There is an interesting but not completely explained link between adipokine production, obesity, and MS, and numerous studies have been conducted in recent years on the level of adipokines in the blood or CSF, establishing a possible correlation with different forms of MS, their evolution, and their disability status. Adipokines may thus play a role in potential biomarkers, both for the early detection of the disease and for the monitoring of the disease activity under chronic disease-modifying drug therapy.

Leptin is the most studied adipokine. Leptin is a peptide of 167 amino acids and a molecular weight of 16 kD secreted mainly from white adipose tissue, with the role of controlling food consumption and catabolism, a function performed by communication with the CNS through specific receptors (Dragano et al., 2017) [36]. Leptin upregulates pro-inflammatory cytokines, such as TNF-alpha and IL-6, and acts directly on macrophages to increase their phagocytic activity and pro-inflammatory cytokine production and also exerts an effect on T cells (Maurya et al., 2018) [37]. The potent modulating effect on the immune response is worth mentioning, with leptin being upregulated by inflammatory mediators. 

Circulating leptin levels are directly correlated with adipose tissue mass. However, several recent studies focus on the multicausality of obesity, through various genetic lesions and mostly from the high exogenous caloric intake and sedentary nature of modern society (Izquierdo et al., 2019) [38]. At the same time, new evidence shows blood–brain-barrier (BBB) changes in obesity (probably similar to BBB alterations in MS), promoting the idea that leptin resistance is caused by the affected transport of leptin at BBB level (Pan and Myers, 2018) [39]. This pathophysiological theory is supported by clinical results, with a relevant example being the study of Keyhanian et al., 2019, that showed an increased level of leptin in patients with pediatric MS [40]. 

As a cytokine that promotes satiety, its function is altered in the case of obesity, a phenomenon called “leptin resistance” (Liu et al., 2018) [41]. Recent studies suggest that elevated leptin levels in serum and CSF in RRMS patients (Evangelopoulos et al., 2014) [42]. This determination correlates with elevated levels of other proinflammatory adipokines such as resistin or adipsin and an increased immune cell activity (upregulation of Th1 and Th17 cells). Initial studies have been performed on the EAE murine model; an example is a study conducted by (Hsuchou et al., 2013), which demonstrated an upregulated leptin receptor expression in areas with pathological activity of the CNS, along with an intense transport in the hippocampal and cervical regions [43]. Moreover, leptin was found in high amounts in serum, especially in cases of active, more severe disease forms. The correlation with immune cells was also studied, with Ouyang et al., 2014, demonstrating on the EAE model that the removal of leptin receptors meant an attenuation of leukocyte infiltration at the CNS level and the reduction in BBB alterations [44]. 

Matarese et al., 2010, were the first to mention the link between elevated leptin levels in CSF and serum of RRMS patients [45]. Subsequently, (Yousefian et al., 2018), in an attempt to provide a pathophysiological explanation, showed that leptin gene polymorphism is correlated with leptin levels and MS, which are both gender-dependent [46]. Finally, Marrodan et al., 2021, confirmed the direct relationship between leptin levels and obesity in MS patients, with leptin also negatively correlating with low levels of Treg cells, whose proliferation is inhibited [47].

Adiponectin (APN), an adipokine with a dual pro- and anti-inflammatory role, and whose plasma level depends on inflammation and the presence of an autoimmune pathology, has been investigated in both mouse and human models. It has been shown that the absence of APN in mice with EAE leads to greater activation of lymphocytes, which means a high severity of the disease (Piccio et al., 2013) [48]. In humans, APN and its associated high level of molecular oligomers when recorded at an elevated level (including in CSF) at the time of diagnosis of MS has a higher risk of disease progression, severity, and disability (Signoriello et al., 2019) (Signoriello et al., 2021) [49,50]. However, discussions remain open, with other studies such as the one conducted by (Baranowska-Bik et al., 2020) finding no correlation between plasma concentration of APN and newly diagnosed MS [51]. Similar results were found in the clinical trial conducted by (Kvistad et al., 2018), in which no statistically significant correlations were found between serum APN or leptin levels and MS activity or response to treatment [52]. 

Resistin, also produced in the CNS by the pituitary gland, could be a future biomarker for MS monitoring, given the increasingly complex correlations between chronic inflammation specific to obese patients and MS, where a correlation between BMI and EDSS has been found (Stampanoni Bassi et al., 2020) [53]. Various studies have shown a high serum resistin level in MS patients compared to the control group, along with significant increases in TNF alpha, IL-1B, or CRP (Hossein-Nezhad et al., 2013) [54]. In addition, elevated resistin levels correlate with decreased Treg lymphocyte activity (Foxp3 nTreg transcription factor) in patients with SMRR (Kraszula et al., 2012) [55].

Adipocyte fatty acid-binding protein (A-FABP), not as intensively studied as the other cytokines presented so far, has been shown to have elevated serum levels in patients with pediatric-onset MS (Messina et al., 2013) [56]. It can be considered a possible marker useful to explain the evolution of the disease over time, proof that A-FABP is increased in secondary progressive multiple sclerosis (SPMS).

Chemerin is expressed in the CNS in vascular endothelial cells and in white matter lesions in MS. Elevated plasma levels have been reported in obese or overweight patients with MS compared to normal-weight patients with MS (Tomalka-Kochanowska et al., 2014) [57]. Newer studies have not replicated the same results, so this correlation remains to be deepened (Koskderelioglu et al., 2020) [58]. As a pathophysiological influence, chemerin is thought to stimulate cell infiltration into the CNS, favoring a more severe form of the disease, but studies are only just in the beginning phase.

For adipsin, although its exact function in relation to metabolism remains unknown, it has been quantified in the serum and CSF of MS patients. In the case of a cohort of Finnish patients, high serum and CSF levels of adipsin were identified (Hietaharju et al., 2010) [59]. Opinions remain divided as to whether serum adipsin levels correlate directly with CSF levels, with no evidence in the Finnish cohort. However, other studies suggest a correlation between plasma and CSF adipsin levels, and these two values are correlated independently of sex, with high values in overweight and obese people (Schmid et al., 2016) [60].

There are other adipokines (visfatin, omentin, vaspin), and although sufficient data are currently lacking, there are high chances that they will become reliable biomarkers for the diagnosis and monitoring of MS, at least in obese patients. New studies on animal models and humans are needed in order to better explain adipokines action and influences in MS. 

Obesity also leads to decreased bioavailability of vitamin D regardless of age or ethnicity, and hypovitaminosis D is correlated with a proinflammatory status (Walsh et al., 2017) [61]. Numerous studies on this topic were conducted in the past, and research continues in the present due to sometimes contrasting results (Popescu et al., 2015) (Sintzel et al., 2018) [62,63]. Some authors suggest that hypervitaminosis D could be protective against relapses (Pierrot-Deseilligny and Souberbielle, 2017) [64], while other trials have not found a significant correlation between serum vitamin D levels and the risk of developing/worsening MS (Jagannath et al., 2018) [65]. 

### 4.3. The Role of the Intestinal Microbiome

Another aspect growing in importance that has been studied intensely in recent years is the relationship between changes in the intestinal microbiome and alterations in the CNS. In the case of MS, the microbiota, depending on the dominant types of bacteria, has various effects. According to Chu et al., 2018, the selectivity of BBB is maintained by a favorable intestinal microbiota that also limits the pathogenicity of astrocytes and can activate microglia [66]. In MS, there is an abundance of certain bacterial species (Archaea), simultaneously with the decrease to the absence of other types such as Firmicutes and Bacteroidetes phyla (Tremlett et al., 2017) [67]. Associations have also been observed between certain microbiota profiles (i.e., Fusobacteria) and an increased risk of recurrent relapses (Buscarinu et al., 2017) [68]. Moreover, it seems that immunomodulatory treatment also modifies the composition of the microbiota: Adamczyk-Sowa et al., 2017, proved the restoration of the physiological order of Bacteroidaceae, Faecalibacterium, Ruminococcus, Lactobacillaceae, and Clostridium following appropriate treatment with glatiramer acetate [69]. We should also mention early research related to probiotic treatment and fecal microbial transplantation that modifies the intestinal microbiota ecosystem and could be useful in MS, although currently without significant therapeutic impact (Bron et al., 2017) [70].

Gut microbiota influences other organs (including CNS) through degradation products obtained after fermentation, with the most important being short-chain fatty acids (SCFAs). Their role in normal and pathological conditions has been the subject of numerous studies; e.g., Dalile et al., 2019 illustrating the existence of at least four pathways of action (immune, endocrine, vagal, and humoral) through which SCFA influences the brain [71].

Modification of the intestinal microbiota means the production of certain substances by fermentation, and these microbiota-specific products have different impacts on the immune system. According to (Cekanaviciute et al., 2017), in patients with MS, there is a particular type of microbiota that will modulate the immune response by inhibiting Treg cells and exacerbating the proinflammatory action of Th1 lymphocytes [72].

Based on these considerations, more studies in humans have followed, and in the case of MS, propionic acid seems to modulate the activity of the disease. Given that low concentrations of propionic acid were found in the blood and feces of MS patients, Duscha et al., 2020 administered exogenous propionic acid as an add-on therapy in a study group, with immunological and disease activity monitoring. Recording a decrease in Th1 activity and an important augmentation of Treg function with less disability and reduced relapse rate, preliminary results are encouraging as more research needs to be done in this direction [73].

### 4.4. The Role of Nutrition

Nutrition may have a double influence on MS disease course. Firstly, with restricted or excessive intake of certain nutritional principles, macro- and micronutrient imbalances can occur at the CNS level, subsequently influencing the inflammatory and demyelinating processes. One good example is the beneficial effects of carotenoids and polyphenols from vegetables in patients with MS (Riccio & Rossano, 2015); however, many other molecules may have antioxidant properties sustaining an anti-inflammatory microenvironment at BBB and CNS levels [74]. Another extensive discussion refers to the role vitamins play in MS modulation, with adequate nutrition being the central source for ensuring optimal vitamin D or vitamin B12 levels (Bagur et al., 2017) [75].

On the other hand, as gut microbiota seems to be a central key player in MS progression, interventions at this level may theoretically alter the pathological immune response and subsequently the disease course. Different diets (caloric restriction, McDougall diet, Mediterranean diet), which may alter gut microbiota, were tested in both clinical and preclinical conditions; however, no consensus has yet been reached. Among the most relevant and recent findings, we mention the work of Sanchez et al., 2020, which illustrates in detail the influence of different dietary restrictions on the components of gut microbiota and offers future directions in diet plans that increase microbiota populations (e.g., Bacteroides) that are normally decreased in MS patients [76].

### 4.5. Obesity Related to Disability and Mortality in MS Patients 

The association between obesity, disability (high EDSS score), and mortality risk is an important topic for the specialist treating MS patients. After a careful search of the literature, the authors found relevant data on this topic published mostly during the last COVID-19 pandemic year. 

The current COVID-19 pandemic has brought new challenges for neurologists and also a need for reanalysis of the criteria on mortality in patients with MS, with emphasis on severe acute respiratory syndrome and hospitalization rate. However, given the short period since the onset of the pandemic, little research has been conducted in this direction, with mainly observational studies on relatively small cohorts of patients. The most significant results are summarized in Table 3. 

## 5. Conclusions and Future Perspectives

Obesity is frequently associated with MS, especially obesity in adolescence, which is associated with an increased risk of both adult and pediatric MS, regardless of gender, with small variations depending on the geographical area. 

Although great progress has been made in elucidating the pathophysiological associations between obesity and MS, these intertwined theories still present aspects that require further research. As obesity in MS patients becomes increasingly important for neurologists, researchers are trying to find other related features that may also act as a starting point for future therapeutic approaches. One good example is the influence of diet on the disease onset, progression, and symptomatology; some preclinical models and epidemiologic and limited prospective studies provide only preliminary evidence [82]. With many different dietary patterns available (caloric restriction, McDougall diet, Mediterranean diet), and knowing that environmental factors can influence MS course, further research on this topic is currently ongoing. 

Finally, this review opens a new perspective of research, mainly regarding the unknown pathophysiological elements of the obesity–MS epidemiological correlation. In light of the new COVID-19 pandemic context, obesity in MS patients has become of much importance, with an independent influence on the severity and outcome of SARS-CoV-2 acute respiratory insufficiency. Furthermore, new large-cohort studies are needed to understand the impact of obesity on morbidity, mortality, and disability of MS patients who contracted COVID-19 vs. those who did not. 

## Figures and Tables

**Table 1:** Recent findings on the association between obesity and MS in both adult and pediatric patients.

Reference	Study Population MS Cases vs. (Control Cases)	Exposure (Childhood vs. Adolescent vs. Adult Obesity)	Study Outcome (Early-Onset MS vs. MS vs. MS Progress)
Mendelian randomization studies			
Mokry et al., 2016 [13]	14,498 (346,196)	Childhood and/or early adulthood obesity	MS in adults
Gianfrancesco et al., 2017 [20]	569 (16,820)	Childhood obesity	Pediatric MS
Harroud et al., 2021 [14]	14,802 (26,703)	Childhood obesity	MS in adults
Epidemiological studies			
Langer-Gould et al., 2013 [15]	75	Childhood obesity	MS/CIS in adolescent girls
Kvistad et al., 2015 [19]	86	Adult obesity	MS progress
Chitnis et al., 2016 [16]	254 (420)	Adolescent obesity	Pediatric MS
Hedstrom et al., 2016 [12]	1586 (2800)	Childhood and adolescent obesity	MS in adults
Huppke et al., 2019 [18]	453	Childhood and adolescent obesity	Pediatric MS MS progress

**Table 2:** Main findings on the influence of geographical area on the obesity–MS association extracted from recent studies.

Reference	Number of MS Patients	Female: Male Ration	Disease Duration Mean, y	Geographical Area	Obesity in MS Group (vs. Control Group)	*p*-Value
Sicras-Mainar et al., 2017 [22]	222	143:79	13.4 (9.5)	Asturia and Catalonia, Spain	22.5%	0.935
Sakoda et al., 2020 [23]	103	80:23	13 (8.6)	Kyushu Island, Japan	19.4% (vs. 7.4%)	0.009
Fahmi et al., 2020 [24]	60	41:19	4.25 (3.89)	Egypt	53.3%	0.533
Soliman et al., 2020 [25]	50	34:16	7.3 (5.1)	Cairo, Egypt	38% (vs. 0%)	<0.001

**Table 3:** Impact of obesity on disability in MS patients—focus on COVID-19.

Reference	Study Population			Modified COVID-19 Mortality Risk Score > 3 or Hospitalization	Obesity in MS Group
	Number of MS Cases	Female: Male Ratio	Disease Duration, Mean, (SD), y		
Bsteh et al., 2020 [77]	1931	1394:537	15	9.7%	12.2%
Louapre et al., 2020 [78]	347	249:98	13.5 (10)	21%	6.9%
Mohn et al., 2020 [79]	873	-	10.7 (7.8)	29%	17.6%
Parrotta et al., 2020 [80]	76	46:30	15.2 (10.7)	23.6%	30.2%
Zabalza et al., 2020 [81]	93	62:31	15.1 (4.5)	20.4%	26.3%

## Data Availability

The authors declare that data supporting the findings of this study are available within the article.

## Abbreviations

BBB	Blood–brain barrier
BMI	Body mass index
CNS	Central nervous system
CSI	Clinically isolated syndrome
IL-6	Interleukin 6
MetS	Metabolic syndrome
MS	Multiple sclerosis
NEDA	No evidence of disease activity
TNFalpha	Tumor necrosis factor alpha
